# Dynamics of Adrenal Steroids Are Related to Variations in Th1 and Treg Populations during *Mycobacterium tuberculosis* Infection in HIV Positive Persons

**DOI:** 10.1371/journal.pone.0033061

**Published:** 2012-03-14

**Authors:** Maria Florencia Quiroga, Matias Tomas Angerami, Natalia Santucci, Diego Ameri, Jose Luis Francos, Jorge Wallach, Omar Sued, Pedro Cahn, Horacio Salomón, Oscar Bottasso

**Affiliations:** 1 Department of Microbiology, National Reference Center for AIDS, University of Buenos Aires School of Medicine, Buenos Aires, Argentina; 2 Institute of Immunology, University of Rosario School of Medical Sciences, Rosario, Argentina; 3 Infectious Diseases Unit, J.A. Fernández Hospital, Buenos Aires, Argentina; 4 Huesped Foundation, Buenos Aires, Argentina; 5 21 Unit, F.J. Muñiz Infectious Diseases Hospital, Buenos Aires, Argentina; Statens Serum Institute, Denmark

## Abstract

Tuberculosis (TB) remains the most frequent cause of illness and death from an infectious agent, and its interaction with HIV has devastating effects. We determined plasma levels of dehydroepiandrosterone (DHEA), its circulating form DHEA-suphate (DHEA-s) and cortisol in different stages of *M. tuberculosis* infection, and explored their role on the Th1 and Treg populations during different scenarios of HIV-TB coinfection, including the immune reconstitution inflammatory syndrome (IRIS), a condition related to antiretroviral treatment. DHEA levels were diminished in HIV-TB and HIV-TB IRIS patients compared to healthy donors (HD), HIV+ individuals and HIV+ individuals with latent TB (HIV-LTB), whereas dehydroepiandrosterone sulfate (DHEA-s) levels were markedly diminished in HIV-TB IRIS individuals. HIV-TB and IRIS patients presented a cortisol/DHEA ratio significantly higher than HIV+, HIV-LTB and HD individuals. A positive correlation was observed between DHEA-s and CD4 count among HIV-TB individuals. Conversely, cortisol plasma level inversely correlated with CD4 count within HIV-TB individuals. *M. tuberculosis*-specific Th1 lymphocyte count was increased after culturing PBMC from HIV-TB individuals in presence of DHEA. We observed an inverse correlation between DHEA-s plasma level and Treg frequency in co-infected individuals, and CD4+FoxP3+ Treg frequency was increased in HIV-TB and IRIS patients compared to other groups. Strikingly, we observed a prominent CD4+CD25-FoxP3+ population across HIV-TB and HIV-TB IRIS patients, which frequency correlated with DHEA plasma level. Finally, DHEA treatment negatively regulated FoxP3 expression without altering Treg frequency in co-infected patients. These data suggest an enhancing role for DHEA in the immune response against *M. tuberculosis* during HIV-TB coinfection and IRIS.

## Introduction

Tuberculosis continues to be the most successful pathogen around the world, and its interaction with HIV fuels both epidemics [1]. Infection largely takes place in the lungs and most newly infected persons experience a favourable course of these lesions with the caseating necrotic material being encapsulated by concentric layers of epithelioid cells, lymphocytes and some giant cells. In a few cases granulomas may evolve unsatisfactorily with softening of the caseum and presence of very high numbers of bacilli which are expelled through the bronchus or spread via the hematogenous route. Post-primary TB can result in distinct clinical outcomes starting with asymptomatic latent infection, through mild forms of disease, to progressive disease with important necrosis and cavity formation [2]. The great majority of TB cases result from reactivation of old lesions, likely due to the presence of predisposing factors such as malnutrition, steroid therapy or concomitant diseases, i.e., diabetes, leukaemia or HIV infection [3], [4]. Tuberculosis and HIV are two chronic infectious diseases capable of influencing each other throughout several immune-dysregulated processes. For example, HIV infection alters the immune response to mycobacteria, notably *Mycobacterium tuberculosis*, with significant morbidity and mortality in HIV-positive patients. The introduction of the highly active antiretroviral therapy (HAART) to treat HIV infection resulted in the restoration of the immune system an improvement of immunity against HIV and other pathogens [5]. In some HIV-infected patients the restoration of pathogen-specific immune responses during the initial months of highly active antiretroviral treatment (HAART) could elicit immunopathological inflammatory responses, leading to the development of immune reconstitution inflammatory syndrome (IRIS). The prevalence of IRIS among HIV patients starting HAART ranges from 3 to 31.7% and includes viral, fungal, parasitic and bacterial diseases [6]. In the case of TB, IRIS manifests either as paradoxical TB IRIS (a clinical deterioration during specific antibiotic therapy), or as unmasking TB IRIS (a clinical manifestations of latent TB infection). Mycobacteria account for approximately 40% of all cases of infectious IRIS in patients initiated on HAART [7], [8]. The frequency of paradoxical TB-IRIS range from 8% to 43% whereas the occurrence of unmasking TB IRIS is more restricted [6]. As occurs in most infectious diseases, clinical manifestations of TB and IRIS is the result of a complex series of interactions between pathogens and immune response [6].

The demonstration of an increase in the number of purified protein derivative-specific Th1 IFN-γ-producing T cells and Th1 cytokines in IRIS patients [9] and a study in Africa employing several mycobacterial antigens [10] point out a role of Th1 cells as being implied on IRIS phenomenon. Moreover, the relevance of Th1 responses in tuberculosis has been very well described. Recently, it has been demonstrated for the first time that IFN-γ combined with vitamin D are able to kill *M. tuberculosis* inside infected human macrophages, showing the relevance of endocrine modulation on anti-mycobacterial immune responses [11].

Besides the intrinsic immunomodulatory influences accounting for disease occurrence, factors like steroid hormones are likely to play a role in this regard. In fact, inflammatory cytokines produced during tuberculosis, i.e., IFN-γ, TNF-α and IL-6 can also activate the hypothalamic-pituitary-adrenal (HPA) axis leading to the final production of steroid hormones by the adrenal with well known influences on the immune response [12]. As an example, glucocorticoids (GC) can promote a Th2 cytokine acquisition profile [13], facilitating Th2 activities, whereas its natural antagonist dehydroepiandrosterone -DHEA- is able to favour Th1 cytokine production and interfere with Th2 cytokine synthesis [14]. DHEA and its sulfated prohormone, DHEA sulfate (DHEA-s) are quantitatively the most abundant circulating adrenal steroid hormones in humans. Circulating DHEA-s serves as a reservoir for DHEA, with conversion by sulfotransferases occurring in a wide range of tissues. There is also extensive metabolism to estrogens and androgens, giving rise to the view that many of its effects are mediated by these hormones or other metabolites like androstenediol and androstenetriol [15]. Whereas DHEA-s is the hydrophilic storage form that circulates in the blood, only lipophilic DHEA can be converted intracellularly to androgens and estrogens. Thus, the tissue-specific synthesis of DHEA sulfotransferase and steroid sulfatase determines the ratio of DHEA activation (by conversion to sex steroids) to transient DHEA inactivation (by secretion of the sulfate ester back into the bloodstream) [15], [16]. Working in conjunction, all these factors may modify the nature of the specific immune response that underlies the spectrum of clinical manifestations during HIV-TB infections. Regarding the utilization of DHEA and/or DHEA derivatives in the treatment of tuberculosis, it has been shown that the synthetic adrenal steroid derivative 16 α-bromoepiandrosterone (EpiBr; 16 α-bromo-5α -androstan-3β-ol-17-one), exerted beneficial effects in clinical and experimental tuberculosis and HIV patients [17], [18], [19].

Regulatory T cells (Treg) play a central role in the prevention of autoimmunity and in the control of immune responses by down-regulating the function of effector CD4(+) or CD8(+) T cells. Moreover, Treg are central for maintaining the balance between immune-mediated suppression of *M. tuberculosis* and immunopathology in patients with tuberculosis [20], [21]. Given the role of Treg in the maintenance of immune homeostasis quantitative or functional defects in these cells may be also involved in TB-IRIS [22].

Within this setting our study sought to investigate the immuno-endocrine interactions occurring during different clinical scenarios of HIV-TB co-infection as an attempt to get a better understanding of the disease physiopathology. Specifically we aimed to explore the role of adrenal hormones in the modulation of the frequency of *M. tuberculosis* - specific Th1 lymphocytes and Treg during co-infection with HIV and TB and also during the course of IRIS. We hypothesize that cell frequency will be influenced by cortisol and DHEA, modulating both innate and adaptive immune responses to *M. tuberculosis*.

## Materials and Methods

### Patients and study design

The present study has a cross-sectional design in which patients were included prospectively during a 3-year period. Five groups of individuals were enrolled: 1) Asymptomatic HIV-1 infected patients (HIV+, n = 10, male: 6, female: 4), as determined by ELISA and confirmatory Western Blot who were tuberculin skin test negative (TST^neg^); 2) HIV-1 infected patients with active TB, who received less than one week of anti-TB therapy also TST^neg^ (HIV-TB, n = 21, male: 17, female: 4); 3) HIV-TB individuals with TB-IRIS (for simplicity, denoted as IRIS patients from now on, n = 6, male: 4, female: 2); 4) HIV-1 infected individuals latently infected with *M. tuberculosis* (HIV-LTB, n = 5, male: 2, female: 3) as determined by TST and 5) Bacillus Calmette-Guerin-vaccinated healthy donors TST^neg^ (HD, n = 16, male: 8, female: 8). No patient received DHEA and/or glucocorticoid treatment. Comparisons by sex yielded no differences in the immune-endocrine variables under analysis. Patients were evaluated at Hospital J.A. Fernández and Hospital F.J. Muñiz, Buenos Aires, Argentina. Diagnosis of TB was based on the identification of acid-fast bacilli in sputum, or a positive culture of tuberculosis bacilli and clinical and/or radiological data. All HIV individuals were under antiretroviral treatment following current guidelines [23]. HIV and HIV-TB patients had no evidence of IRIS development. IRIS was diagnosed following the criteria proposed by International Network for the Study of HIV-associated IRIS [10]. All IRIS cases were first diagnosed as having TB and afterwards initiated HIV treatment; therefore diagnosed as paradoxical TB. Written informed consent was obtained, according to the local Ethics Committee. The characteristics of all participants are shown in Table 1.

**Table 1:** Epidemiological characteristics of the subjects enrolled.

	Active TB	Latent TB	IRIS	HIV	HD
	*n* 21	*n* 5	*n* 6	*n* 10	*n* 16
Median age in years (IQR)	35 (32 – 45)	36 (29 – 47)	31.5 (30 – 34)	37 (33.5 – 41)	39 (22 – 42)
Female/male distribution	4/17	3/2	2/4	4/6	8/8
HIV status (positive over total)	21 (100%)	5 (100%)	6 (100%)	10 (100%)	0 (0%)
Median CD4 count (IQR)	53 (25.4 – 887)	541(505 – 576)	199.8 (54.41 – 255.1)	433.5 (189 – 588.8)	769 (602 – 887)
Median Viral Load (IQR)	78999 (986.5 – 389992)	138 (65 – 21777)	380,5 (187.8 – 1146)	71,5 (50 – 1377)	N/A
BCG vaccinated (positive over total)	21 (100%)	5 (100%)	6 (100%)	10 (100%)	16 (100%)

BCG, Bacillus Calmette-Guerin. IQR, Interquartile range.

### Hormone assessments

In order to assess cortisol, DHEA and DHEA-s plasma levels EDTA-anticoagulated blood samples were drawn. After centrifugation, plasma aliquots were preserved at -80°C. Cortisol was measured by electrochemiluminescence (automatic analyser ADVIA Centaur® XP Immunoassay System, Siemens Healthcare Diagnostics, Deerfield, IL, USA), DHEA by radioimmunoassay (Packard Cobra II Gamma Counter, Packard, Meriden, CT, USA) and DHEA-s by immunochemoluminiscence (IMMULITE® 1000, Siemens Healthcare Diagnostics, Deerfield, IL, USA) tests. All hormone determinations were performed in the same blood samples collected between 8:00 and 8:30 a.m. because of the circadian rhythms.

### Antigen

*In vitro* stimulation of cells was performed with pre-titrated heat-killed *M. tuberculosis* H37Rv (Mycobacteria Research Laboratories, Colorado State University, Fort Collins, CO) at 10 µg/ml final concentration for all the experiments prepared by probe sonication.

### ELISPOT assays

IFN-γ-secreting cells were detected using enzyme-linked immunospot (ELISPOT) assays conducted as described previously [24]. Briefly, peripheral blood mononuclear cells (PBMC) were cryopreserved as described elsewhere [24], and 1 day before the assay, they were thawed and rested overnight in RPMI medium supplemented with 10% fetal bovine serum (Gibco BRL), 2 mM Lglutamine (Gibco BRL), 100 U/ml penicillin (Gibco BRL), 100 mg/ml streptomycin (Gibco BRL), and 10 mM HEPES (Gibco BRL) and viability was checked by trypan blue exclusion, as described [24]. Rested PBMCs were plated on sterile 96-well plates (MultiScreen IP plates; Millipore), previously coated with mouse anti-human IFN-γ monoclonal antibody (BD Biosciences) at 10^5^ cells/well. Heat-killed *M. tuberculosis* strain H37Rv was added in duplicate wells (final concentration, 10 µg/ml). Stimulating dose of *M. tuberculosis* antigen was determined by titration using PBMC from healthy volunteers stimulated *in vitro*. Cortisol or DHEA (both from Sigma-Aldrich) were added at different physiological concentrations of DHEA (10^−7^ M, 10^−8^ M) and a slightly supraphysiological concentration of cortisol (10^−6^ M). Negative (medium plus 0.05% DMSO) and positive (PMA 5 ng/ml plus Ionomicin, 500 ng/ml, both from Sigma-Aldrich) controls were included for each patient. Plates were developed using biotinylated anti-human IFN-γ monoclonal antibody, streptavidin-peroxidase complex, and AEC (3-amino-9-ethylcarbazole) substrate reagent set (BD Biosciences). Plates were scanned on an ImmunoSpot reader (Cellular Technology Ltd.). Specific spots were counted using the ImmunoSpot software. Results were expressed as spot forming units (SFU)/10^6^ PBMC after subtraction of the negative-control values.

### Culture conditions

PBMC were isolated by density gradient centrifugation on Ficoll-Paque and cultured at 1×10^6^ cells/ml with *M. tuberculosis* (10 µg/ml) in 24 or 96-well plates with RPMI medium supplemented with 10% fetal bovine serum (Gibco BRL), 2 mM Lglutamine (Gibco BRL), 100 U/ml penicillin (Gibco BRL), 100 mg/ml streptomycin (Gibco BRL), and 10 mM HEPES (Gibco BRL) in the presence or absence of cortisol and DHEA (both from Sigma-Aldrich) at the indicated concentrations. After 3 days, cells were harvested, and Treg frequency was determined by flow cytometry.

### Treg determination

Freshly isolated PBMC or cultured cells were stained with anti-CD4 PerCP, anti-CD25 FITC (BD Biosciences) for 15 min at 4°C in the dark. Subsequently, they were fixed and permeabilized with the Human FoxP3 Buffer Set (BD Biosciences) following the manufacturer’s instructions, before intracellular staining with PE-conjugated FoxP3 (259D/C7, BD Biosciences) for 30 min. For flow cytometric analysis, lymphocytes were gated on CD4^+^ lymphocytes, and afterwards the percentages of CD25^+^ and/or FoxP3^+^ cells was determined.

### Statistical analysis

Statistical analysis was performed using the non-parametric Wilcoxon matched pairs test for paired samples, and the Kruskall-Wallis analysis of variance, followed by post hoc comparisons (Dunns test) when applicable for between-groups comparisons. Correlations were determined using Spearman’s rank test. Analyses were performed using Prism v 5.01software (GraphPad Software Inc., La Jolla, CA, USA). A *p* value < 0.05 was considered statistically significant.

## Results

### Hormone levels and IFN-γ responses along the spectrum of HIV-TB coinfection

**Figure 1:**
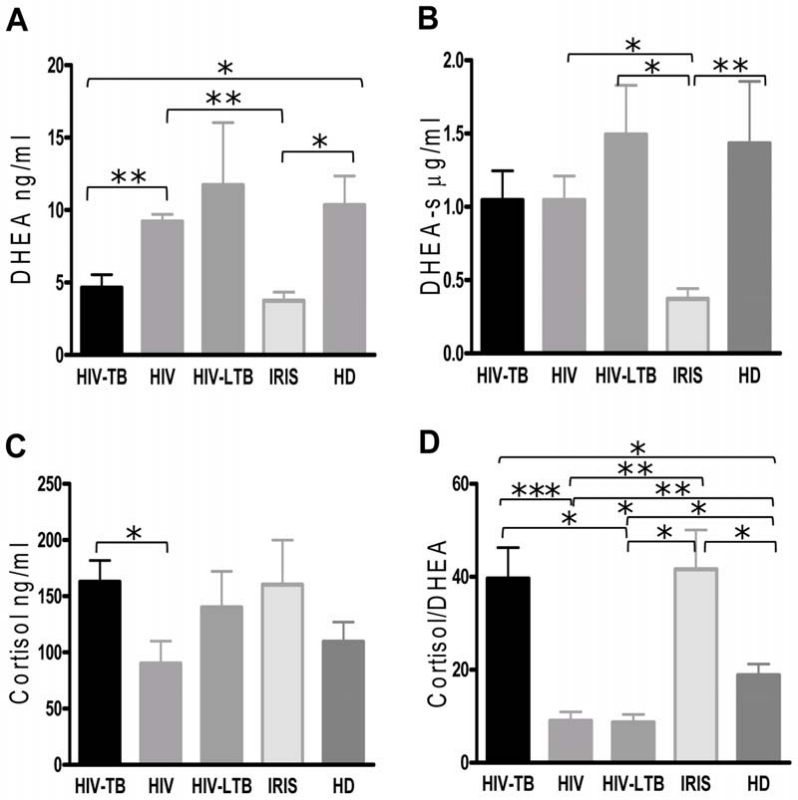
Cortisol, dehidroepindrosterone-sulfate (DHEA-s), DHEA levels and Cortisol/DHEA ratio in HIV, HIV-TB, IRIS, HIV-LTB and HD. **A.** DHEA, **B.** DHEA-s, **C.** Cortisol and **D.** Cortisol/DHEA (ratio) levels in plasma of HIV+, HIV-TB, IRIS, HIV-LTB, and HD individuals. Bars indicate the mean ± SEM for each group. Horizontal lines indicate comparisons between groups and statistically significant differences. DHEA was measured by radioimmunoassay, DHEA-s by immunochemoluminiscence tests and Cortisol by electrochemiluminescence. HIV+ individuals n = 10, HIV-TB n = 21, IRIS n = 6, HIV-LTB n = 5, and HD n = 16. *: *p* < 0.05; **: *p* < 0.01; ***: *p* < 0.001.

In order to determine an association of DHEA, DHEA-s and cortisol levels with HIV-TB, IRIS, HIV+ or HIV-LTB conditions, we initially assessed adrenal hormone levels in plasma from patients and healthy donors (HD). Previously, we observed a profound decrease in DHEA levels in TB patients, in parallel to an increase in cortisol [25], We observed that DHEA plasma levels were significantly diminished in HIV-TB and IRIS patients compared to HD, HIV-LTB and HIV+ individuals (p<0.05), whereas DHEA-s levels were markedly diminished in HIV-TB with IRIS individuals (p<0.01 compared to HD, Fig. 1A y 1B). Furthermore, cortisol values were similar among groups except for HIV+ persons, whose cortisol levels were significantly lower compared to HIV-TB patients (p<0.05, Fig. 1C). When analyzing the cortisol/DHEA ratio, co-infected patients and IRIS patients presented a cortisol/DHEA ratio significantly higher than HIV+, HIV-LTB and HD individuals (p<0.001, p<0.05, p<0.05 respectively, Fig. 1D), with HIV+ and HIV-LTB patients displaying slightly but still statistically significant lower cortisol/DHEA ratio than HD (p<0.01 and p<0.05 respectively, Fig. 1D).

Continuing our studies, we wished to investigate the relationship between immune status and adrenal hormone levels during HIV and tuberculosis co-infection, we performed correlation analyses between DHEA-s or cortisol levels and CD4^+^ T cell counts. A positive correlation was observed between DHEA-s and CD4 counts among HIV-TB individuals (*r_s_* = 0.450195, *p* = 0.0406, *n* = 21), but also when grouping all the individuals regardless their condition (*r_s_ = *0.461295, *p = *0.0027, *n = *44). In contrast, cortisol plasma levels correlated inversely with CD4 counts within HIV-TB individuals (*r_s_* = -0.5160, *p* = 0.0237, *n* = 21), and all the enrolled individuals (*r_s_* = -0.3869, *p* = 0.0066, *n* = 44).

**Figure 2:**
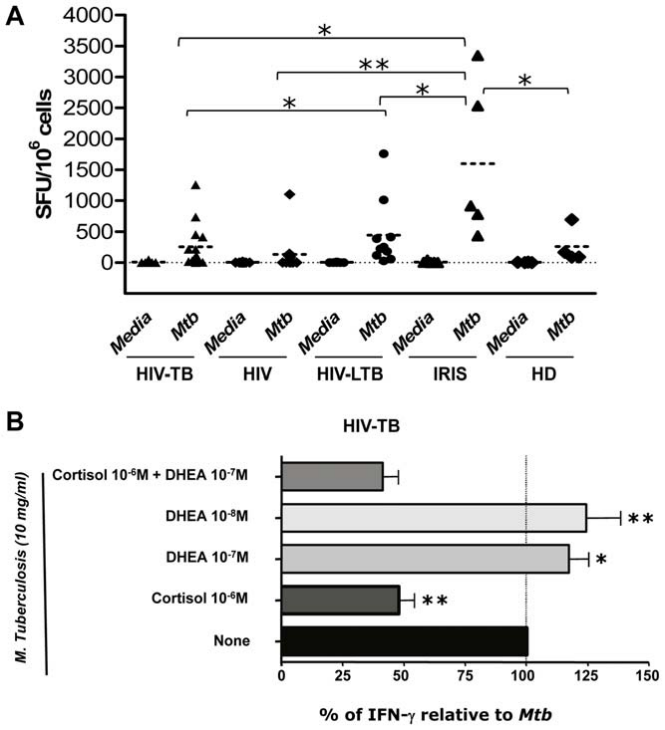
Modulation of *M. tuberculosis*-induced IFN-γ production by adrenal hormones. **A.** Absolute numbers of IFN-γ producer cells from PBMC of HIV+, HIV-TB, IRIS, HIV-LTB and HD patients, which have been stimulated with *M. tuberculosis* antigen for 16 hours. Horizontal lines indicate the mean and comparisons between groups and statistically significant differences are shown. SFU, Spots forming units. **B.** Percentage of IFN-γ producer cells relative to *M. tuberculosis* in PBMC of HIV-TB patients (n = 12). PBMC (10^5^ cells/well) were stimulated in the presence of *M. tuberculosis* with or without addition of DHEA and/or cortisol at the indicated concentrations. Each bar illustrates the mean ± SEM of the percentage for IFN-γ producer cells relative to *M. tuberculosis* for the each group, calculated as follows: % of IFN-γ relative to *Mtb* = ([(*Mtb* hormone-Media)-(*Mtb*-Media)]/(*Mtb*-Media))×100. Asterisks indicate comparisons between each condition against *Mtb* specific response. *: *p* < 0.05; **: *p* < 0.01.

Since DHEA has the potential to induce Th1 responses [25], [26], we aimed to investigate the role of adrenal hormones on the IFN-γ responses to *M. tuberculosis* in co-infected patients. First, we aimed to define each group’s antigen-specific responses by ELISPOT assays and observed an increased frequency of IFN-γ producing cells in IRIS patients compared to HIV, co-infected, latently co-infected and HD individuals (Fig. 2A).

When studying the effect of adrenal hormones on the IFN-γ responses from HIV-TB co-infected individuals, we observed that cortisol treatment decreased the frequency of cytokine-producing cells, and this effect could not be reverted by DHEA addition (Fig. 2B). Notably, DHEA treatment could significantly enhance the IFN-γ responses to the antigen in co-infected patients (Fig. 2B). Moreover, IRIS patients displayed the same trend as co-infected patients, but without significance, likely due to the lower sample size (data not shown). Finally, no enhancing effect of DHEA on IFN-γ was seen when studying cultures from HIV^+^ individuals, HIV-LTB patients or HD (data not shown).

### FoxP3^+^ T cell and FoxP3^+^ CD25^-^ T cell frequencies are increased and correlate with adrenal hormone plasma level in co-infected and IRIS patients

The frequency of Treg (defined as CD25^+^ FoxP3^+^ regulatory CD4 T cells) was reported to be higher both in HIV and tuberculosis infections [27], [28]. Moreover, a role for Treg in IRIS development has been proposed [22]. Nevertheless, during co-infection there are no clear data available on Treg regarding their frequency and function. Adrenal hormones have been proposed to regulate the expression of the FoxP3 gene [29], therefore having a role in regulating immune responses. Initially, we performed correlation analyses in order to investigate the role of adrenal hormones on the frequency of Treg in the context of HIV-TB co-infection. We observed an inverse correlation between DHEA-s plasma levels and Treg frequency (*r_s_* = -0.5073, *p = *0.0114, *n* = 24) in infected individuals.

**Figure 3:**
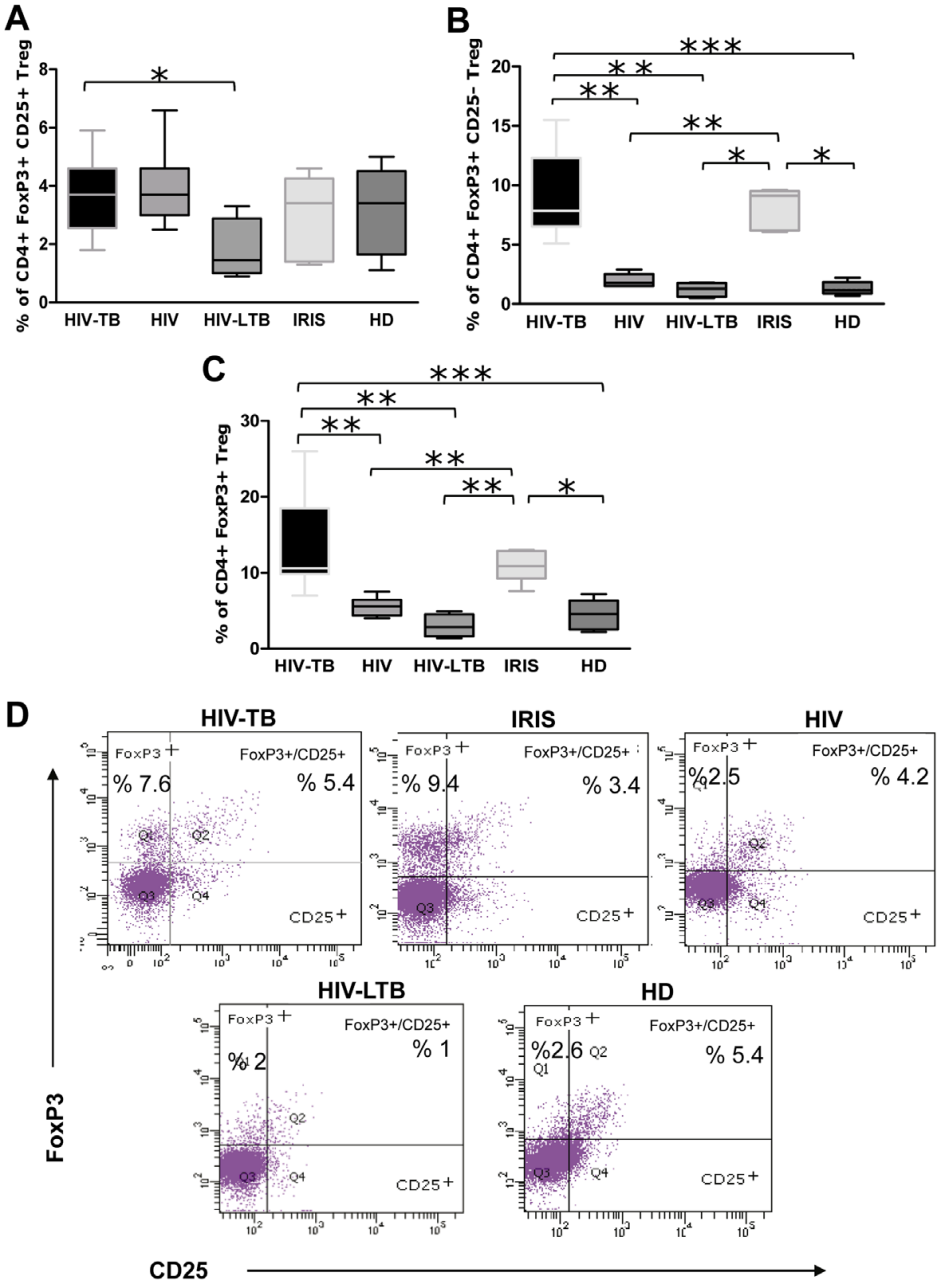
Analysis of Treg population across the spectrum of TB infection. Percentage of Treg cell populations (**A.** CD4+FoxP3+CD25+ Treg, **B.** CD4+FoxP3+CD25- Treg and **C.** CD4+FoxP3+ Treg) of HIV-TB (n = 11), HIV+ (n = 7), HIV-LTB (n = 4), IRIS (n = 5) and HD (n = 10) individuals. **D.** Representative data for Treg flow cytometric analysis from HIV-TB (upper left), IRIS (upper center), HIV (upper right), HIV-LTB (lower left) and HD (lower right) individuals. The plots shown were gated first on lymphocytes by scattering properties, then on CD4+ T cells and finally analyzed for expression of CD25 and FoxP3. **A, B** and **C.** For the box and whisker plots, the horizontal line represents the median, the boxes represent the interquartile range and the whiskers represent the minimum and maximum values. Asterisks indicate comparisons between groups. *: *p* < 0.05; **: *p* < 0.01; ***: *p* < 0.001.

**Figure 4:**
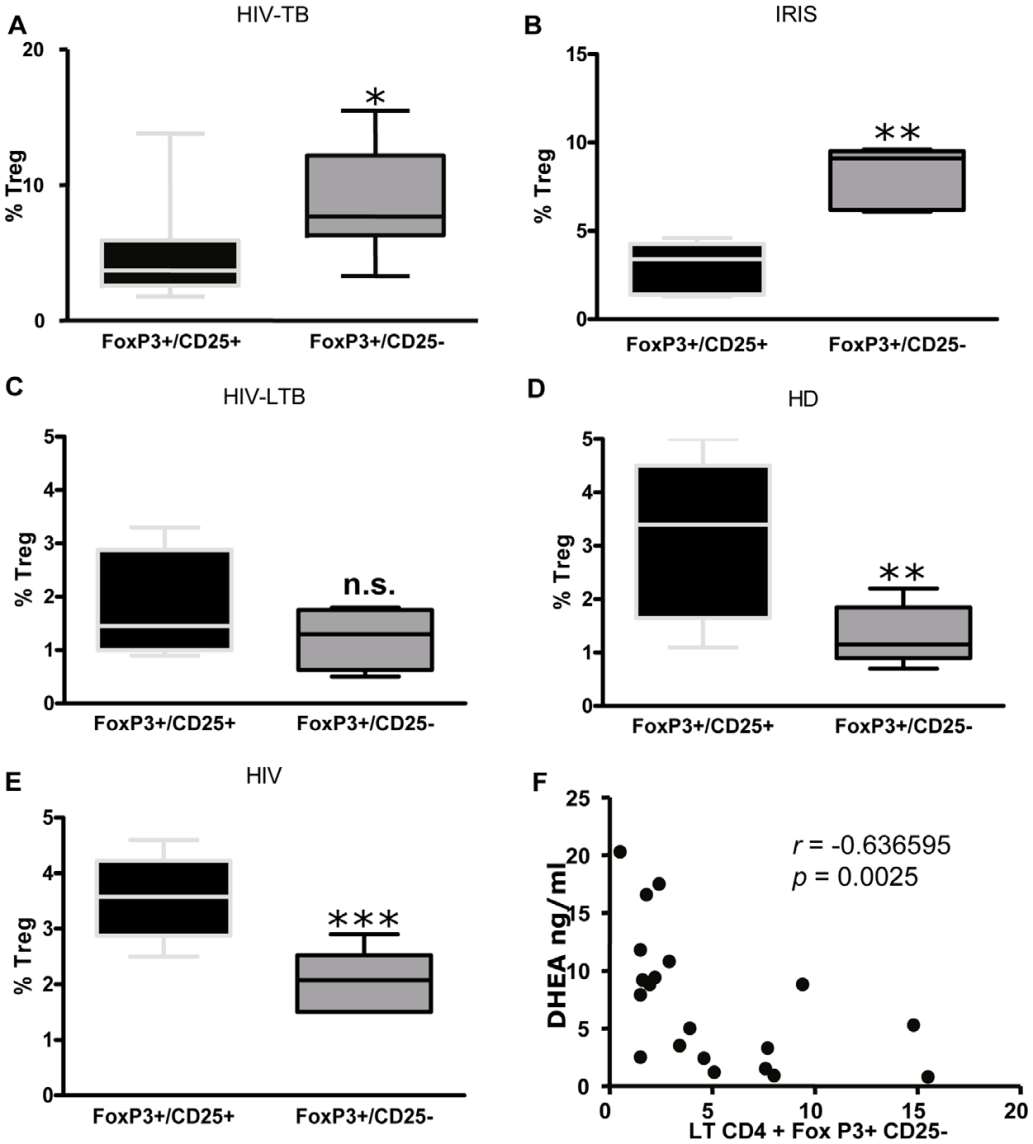
Expanded “non-conventional” Treg in HIV-TB and IRIS patients. Analysis of CD4+FoxP3+CD25+ and CD4+FoxP3+CD25- populations within Treg in **A;** HIV-TB (n = 11), **B;** IRIS (n = 5), **C;** HIV-LTB (n = 4), **D;** HD (n = 10) and **E;** HIV (n = 7) individuals. Horizontal lines inside the boxes indicate the means ± SEM of each group. Asterisks denote comparisons between each sub-population. *: p < 0.05; **: p < 0.01; ***: p< 0.001. **F.** Spearman correlation analysis between plasma DHEA and the percentage of CD4+FoxP3+CD25- lymphocytes from all grouped patients (HIV+, HIV-TB, IRIS, HIV-LTB and HD). Results of statistical analysis are shown in the graphic.

**Figure 5:**
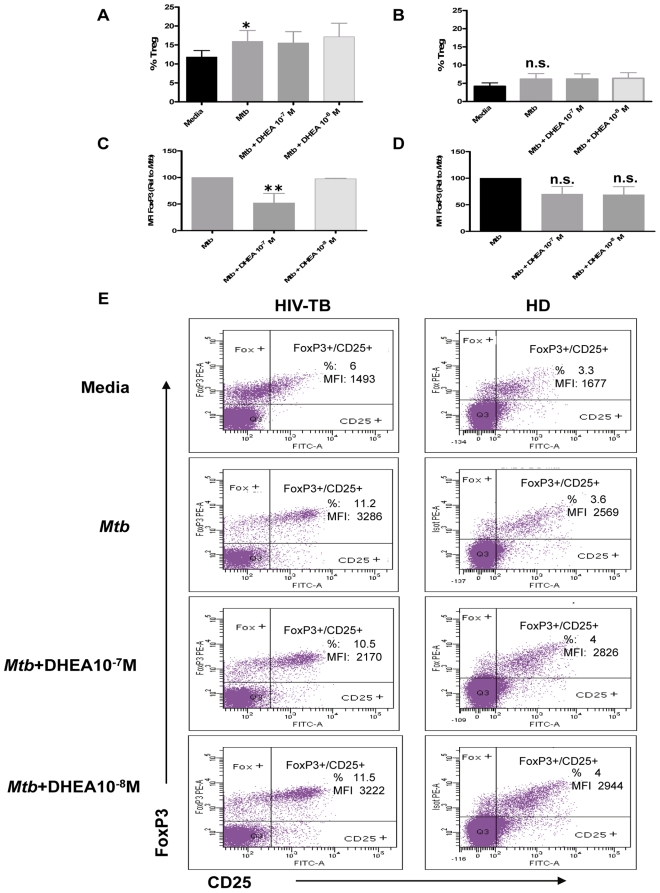
DHEA modulates the expression of the FoxP3 transcription factor in coinfected individuals. A and B. Percentages of Treg cells (defined as CD4+FoxP3+CD25+) in PBMC from **A.** HIV-TB and **B.** HD individuals stimulated with *M. tuberculosis* antigen in the presence or absence of DHEA at the indicated concentrations for 3 days. Bars indicate the mean ± SEM for each experimental condition. **C and D.** Relative FoxP3 Median florescence intensity (MFI) in Treg lymphocytes from **C.** HIV-TB patients and **D.** HD volunteers after culturing PBMC as detailed in A. Bars indicate the mean ± SEM for each treatment. Data are representative of four different experiments. *: *p* < 0.05; **: *p* < 0.01. **E.** Representative flow cytometry graphs depicting the results obtained from culturing PBMC from HIV-TB and HD individuals as indicated above.

When assessing CD4+ CD25+ FoxP3+ Treg frequency, we observed similar percentages among groups, except the HIV-LTB group that showed lower values, statistically significant in relation to HIV-TB patients (Fig. 3A and D). Strikingly, we observed the emergence of a prominent population of Treg lacking CD25 expression among co-infected and IRIS patients (Fig. 3B and D), significantly higher than HIV^+^, HIV-LTB individuals and HD. When analyzing total FoxP3^+^ expression in CD4 T cells irrespective of CD25 expression, we observed the same differences among groups (Fig. 3C and D). We thoroughly studied Treg populations within each group, and observed higher significant proportions of FoxP3^+^ CD25^-^ Treg compared to FoxP3^+^ CD25^+^ in HIV-TB and IRIS patients (Fig. 4A and B), whereas HIV, HIV-LTB individuals and HD showed significantly increased percentages of FoxP3^+^ CD25^+^ Treg compared to FoxP3^+^ CD25^-^ cells (Fig. 4C, D and E). Moreover, the frequency of these “unconventional” FoxP3^+^ CD25^-^ Treg cell negatively correlated with DHEA-s plasma levels (Fig. 5F, *r_s_* = -0.6473, *p* = 0.0006, *n* = 24). Finally, we examined the stability of FoxP3 by culturing PBMC from HIV-TB individuals in the presence of media, with no variation in FoxP3 expression in the CD25 negative population being observed (data not shown).

Finally, we wanted to investigate the role of DHEA on the expression of FoxP3 and the percentage of Treg in co-infected patients. Adding DHEA failed to cause any difference from values seen in cells cultured with control media in either the percentage of FoxP3^+^ CD25^+^ cells or intensity of Foxp3^+^ in those cells (data not shown). *M. tuberculosis* stimulation induced a significant increase of FoxP3^+^ CD25^+^ Treg frequency only in co-infected patients (Fig. 5A) without modifying Treg percentage in healthy donors (Fig. 5B). In both groups, the addition of DHEA resulted in no change in the percentage of Treg cells induced by the antigen. In contrast,, *in vitro* treatment with DHEA significantly diminished FoxP3 expression in FoxP3^+^ CD25^+^ Treg from co-infected patients after culture with *M. tuberculosis* Ag (Fig. 5C and E), without modifying Treg frequency (Fig. 5A and E). On the other hand, culture of PBMC from healthy donors in the presence of DHEA could not modulate the expression of FoxP3 nor the frequency of Treg in response to *M. tuberculosis* stimulation (Fig. 5B, D and E).

## Discussion

People infected with *M. tuberculosis* and HIV exhibit a higher risk to developing progressive diseases from both pathogens. The complex bi-directional interaction between them turns out to favour a *M. tuberculosis*-mediated stimulation of HIV-1 replication, and on the other hand HIV infection exerts detrimental effects on the course of TB. Such scenario is further complicated by the appearance of IRIS during HAART leading to a strong recovery of CD4 T cell responses, which may promote an overt TB or exacerbate a previously established disease.

Data about immuno-endocrine disturbances during HIV-TB co-infection are scarce and our paper provides several pieces of new information about the relationship between the HPA, in terms of adrenal steroids, and the immune response mounted by patients undergoing both infections. Extending previous findings on HIV patients [30] our study demonstrates that DHEA levels are reduced in HIV-TB and HIV-TB-IRIS, the latter group also showing lower amounts of DHEA-s. Reduced levels of DHEA may be the result of a persistent immuno-inflammatory response in both groups of patients. This is in line with a previous report showing the inhibitory effects of culture supernatants from Ag-stimulated PBMC from TB patients on in vitro DHEA production by adrenal cells [12]. The same is likely to apply for the lower amounts of DHEA-s seen in IRIS patients, although some changes in the conversion of DHEA-s into DHEA may be also involved. In this sense, inflammation-related compounds are known to affect DHEA sulphate metabolism [31] and HIV-TB-IRIS is characterized by an increased inflammatory response [32].

Further support for a relative deficiency of the HPA axis during HIV infection is provided by cortisol measurements. In fact, our results showed that HIV patients had rather diminished cortisol levels and HIV-TB and IRIS patients presented modestly increased amounts of this steroid but statistically insignificant in relation to HD. As a result, both groups depicted an increased cortisol/DHEA ratio which may be inefficient for the control of inflammatory reactions, since the slightly increased cortisol levels would not compensate for the anti-inflammatory effect of DHEA [14], because its levels were markedly reduced. Since cortisol is involved with the stress response, whereas DHEA deals with the stress adaptation, the imbalance seen in both groups may reflect a more unfavorable stressful condition which may further impact on the immune responses developed by them.

Our results also expand the knowledge of regulatory influences of adrenal steroids on the immune response. Production of GCs during infections mediates not only anti-inflammatory effects but also favors an immune response that seems to be unsuitable to clear intracellular pathogens [33], [34], [35]. The negative correlation between cortisol levels and CD4 counts in HIV-TB individuals and the inhibitory effects of this hormone on IFN-γ production in the same group of patients is in line with such statement. In contrast, DHEA augmented IFN-γ production in the same group of patients which in turn corroborates its Th1 favoring effects [14].

Dissimilar findings were seen when analyzing correlations. In fact, DHEA correlated positively with CD4 count from HIV-TB patients and the whole population sample, whereas a negative association between DHEA levels and frequency of Treg was found in the former group. This result fits well with the demonstration that DHEA inhibited FoxP3 expression in HIV-TB patients; a finding that provides novel information about the mechanisms by which DHEA modulates the immune response.

In tuberculosis, the potentially protective Th1 cell response may result in an immunopathological response that fails to eliminate the bacteria [36]. Within this setting control mechanisms like those mediated by Treg are essential for preventing immunopathological damage without interfering with processes involved in pathogen clearance [37]. In our series, Treg were only affected in HIV-LTB patients who showed decreased numbers; the implications on whether it represents a different immuno-inflammatory situation affecting both the differentiation and activity of Treg should be analyzed in future studies.

As regards FoxP3, its expression was augmented in HIV-TB and HIV-TB-IRIS patients, particularly in *Mtb*-stimulated CD25+ cells from HIV-TB patients. The transcription factor Foxp3 was originally identified as a master regulator of thymus-derived natural Treg (nTreg) and constitutes the most reliable marker to specify these subsets [37]. Because Foxp3 expression in these Treg is critical for the maintenance of Treg-specific functions [38], it may be assumed that HIV-TB and HIV-TB-IRIS patients have an increased Treg activity likely addressed to ameliorate the accompanying immuno-inflammatory response that usually takes place in these kind of patients**.** Instead of that, an expansion of Treg with a concomitant reduced functional capacity of suppressor cells and diminished IL-10 secretion has been demonstrated in IRIS patients [22], suggesting an underlying defect in these cells, perhaps due to a decreased FoxP3 expression. Other studies in HIV negative individuals with lung TB underline the potential role of Treg since increased frequencies of both total CD4+CD25+ T cells and CD4+ CD25^high^ cells were shown during active disease, preferably in the involved lung [39], [40]. In the same sense, a higher percentage of circulating CD4+CD25^high^ T cells together with increased levels of FoxP3 mRNA in PBMC from TB patients was found, even higher at sites of active inflammation and tissue pathology [41].

In line with the general features of the IRIS, HIV-TB-IRIS patients had a higher IFN-γ production, which among its many activities is known to be essential for the control of *M. tuberculosis* infection [25]. Remarkably, these patients as well as those with HIV-TB also displayed increased levels of FoxP3+ CD25- cells, with reduced numbers of this cell population being found in HIV, HIV-LTB and HD groups. This intriguing cell population is increased in systemic lupus erythematosus patients [42], being its nature under discussion since some authors suggested that these T cells were mostly FoxP3+ CD25- non-Treg [43], whereas others have proposed that these cells were dysfunctional Treg which suppressed T cell proliferation but not IFN-γ production in vitro [44]. FoxP3 expressed by Treg is relatively stable whereas that of non-Treg is not [43], [45]. Therefore, taking into account our observations on the stability of FoxP3, we believe that these cells are dysfunctional Treg. On the other hand, a recent report described a CD25^low^ FoxP3+ GITR+ Treg population in peripheral blood from HD with a strong suppressive activity on T cell proliferation, although the authors didn’t investigate the ability of these cells to inhibit cytokine secretion [46]. Overall, we propose the analysis of these “unconventional” regulatory T cells in HIV-TB co-infection as an interesting issue for future research in the area of tuberculosis pathogenesis in the context of HIV infection.

As recorded in the case of conventional Treg, DHEA concentrations correlated negatively with FoxP3+ CD25- cells. Adrenal hormones modulate the expression of FoxP3 and the frequency of Treg in an autoimmune setting [47], [48]. Thus, we hypothesize that in co-infected patients the increased cortisol to DHEA ratio could upregulate the expression of FoxP3 and therefore the generation of dysfunctional Treg lymphocytes. Our *in vitro* observations support the idea of a negative regulation of the FoxP3 transcription factor by DHEA, which was also seen in cells from Addison’s disease patients [48].

The HPA axis is a major constituent of the neuroendocrine system. Once the magnitude of the defensive response to a microbial agent becomes relevant, activation of the HPA axis follows with significant influences on the immune function [49]. Our results associate adrenal steroids with critical components of the immune response against HIV and *M. tuberculosis*, and provide a stimulating background for studying more about these links, both for a deeper comprehension of pathogenic mechanisms and the potential development of adjuvant interventions.

## References

[1] WHO (2009). Global tuberculosis control: a short update to the 2009 report..

[2] Rook GA, Hernandez-Pando R (1994). T cell helper types and endocrines in the regulation of tissue-damaging mechanisms in tuberculosis.. Immunobiology.

[3] Lawn SD, Zumla AI (2011). Tuberculosis.. Lancet.

[4] Cardona PJ (2010). Revisiting the natural history of tuberculosis. The inclusion of constant reinfection, host tolerance, and damage-response frameworks leads to a better understanding of latent infection and its evolution towards active disease.. Arch Immunol Ther Exp (Warsz).

[5] Guihot A, Bourgarit A, Carcelain G, Autran B (2011). Immune reconstitution after a decade of combined antiretroviral therapies for human immunodeficiency virus.. Trends Immunol.

[6] Calligaro G, Meintjes G, Mendelson M (2011). Pulmonary manifestations of the immune reconstitution inflammatory syndrome.. Curr Opin Pulm Med.

[7] Lawn SD, Bekker LG, Miller RF (2005). Immune reconstitution disease associated with mycobacterial infections in HIV-infected individuals receiving antiretrovirals.. Lancet Infect Dis.

[8] Meintjes G, Lawn SD, Scano F, Maartens G, French MA (2008). Tuberculosis-associated immune reconstitution inflammatory syndrome: case definitions for use in resource-limited settings.. Lancet Infect Dis.

[9] Bourgarit A, Carcelain G, Martinez V, Lascoux C, Delcey V (2006). Explosion of tuberculin-specific Th1-responses induces immune restoration syndrome in tuberculosis and HIV co-infected patients.. Aids.

[10] Meintjes G, Wilkinson KA, Rangaka MX, Skolimowska K, van Veen K (2008). Type 1 helper T cells and FoxP3-positive T cells in HIV-tuberculosis-associated immune reconstitution inflammatory syndrome.. Am J Respir Crit Care Med.

[11] Fabri M, Stenger S, Shin DM, Yuk JM, Liu PT (2011). Vitamin D is required for IFN-gamma-mediated antimicrobial activity of human macrophages.. Sci Transl Med.

[12] Rey AD, Mahuad CV, Bozza VV, Bogue C, Farroni MA (2007). Endocrine and cytokine responses in humans with pulmonary tuberculosis.. Brain Behav Immun.

[13] Bottasso O, Bay ML, Besedovsky H, del Rey A (2007). The immuno-endocrine component in the pathogenesis of tuberculosis.. Scand J Immunol.

[14] Dillon JS (2005). Dehydroepiandrosterone, dehydroepiandrosterone sulfate and related steroids: their role in inflammatory, allergic and immunological disorders.. Curr Drug Targets Inflamm Allergy.

[15] Loria RM (2009). Beta-androstenes and resistance to viral and bacterial infections.. Neuroimmunomodulation.

[16] Rainey WE, Carr BR, Sasano H, Suzuki T, Mason JI (2002). Dissecting human adrenal androgen production.. Trends Endocrinol Metab.

[17] Reading C, Dowding C, Schramm B, Garsd A, Onizuka-Handa N (2006). Improvement in immune parameters and human immunodeficiency virus-1 viral response in individuals treated with 16alpha-bromoepiandrosterone (HE2000).. Clin Microbiol Infect.

[18] Stickney DR, Noveljic Z, Garsd A, Destiche DA, Frincke JM (2007). Safety and activity of the immune modulator HE2000 on the incidence of tuberculosis and other opportunistic infections in AIDS patients.. Antimicrob Agents Chemother.

[19] Hernandez-Pando R, Aguilar-Leon D, Orozco H, Serrano A, Ahlem C (2005). 16alpha-Bromoepiandrosterone restores T helper cell type 1 activity and accelerates chemotherapy-induced bacterial clearance in a model of progressive pulmonary tuberculosis.. J Infect Dis.

[20] Cooper AM (2009). T cells in mycobacterial infection and disease.. Curr Opin Immunol.

[21] Chen X, Zhou B, Li M, Deng Q, Wu X (2007). CD4(+)CD25(+)FoxP3(+) regulatory T cells suppress Mycobacterium tuberculosis immunity in patients with active disease.. Clin Immunol.

[22] Seddiki N, Sasson SC, Santner-Nanan B, Munier M, van Bockel D (2009). Proliferation of weakly suppressive regulatory CD4+ T cells is associated with over-active CD4+ T-cell responses in HIV-positive patients with mycobacterial immune restoration disease.. Eur J Immunol.

[23] OARAC (2011). http://www.aidsinfo.nih.gov/ContentFiles/AdultandAdolescentGL.pdf.

[24] Turk G, Gherardi MM, Laufer N, Saracco M, Luzzi R (2008). Magnitude, breadth, and functional profile of T-cell responses during human immunodeficiency virus primary infection with B and BF viral variants.. J Virol.

[25] Bozza VV, D’Attilio L, Mahuad CV, Giri AA, del Rey A (2007). Altered cortisol/DHEA ratio in tuberculosis patients and its relationship with abnormalities in the mycobacterial-driven cytokine production by peripheral blood mononuclear cells.. Scand J Immunol.

[26] Araghi-Niknam M, Liang B, Zhang Z, Ardestani SK, Watson RR (1997). Modulation of immune dysfunction during murine leukaemia retrovirus infection of old mice by dehydroepiandrosterone sulphate (DHEAS).. Immunology.

[27] Bi X, Suzuki Y, Gatanaga H, Oka S (2009). High frequency and proliferation of CD4+ FOXP3+ Treg in HIV-1-infected patients with low CD4 counts.. Eur J Immunol.

[28] Garg A, Barnes PF, Roy S, Quiroga MF, Wu S (2008). Mannose-capped lipoarabinomannan- and prostaglandin E2-dependent expansion of regulatory T cells in human Mycobacterium tuberculosis infection.. Eur J Immunol.

[29] Xiang L, Marshall GD (2011). Immunomodulatory effects of in vitro stress hormones on FoxP3, Th1/Th2 cytokine and costimulatory molecule mRNA expression in human peripheral blood mononuclear cells.. Neuroimmunomodulation.

[30] Christeff N, Gherbi N, Mammes O, Dalle MT, Gharakhanian S (1997). Serum cortisol and DHEA concentrations during HIV infection.. Psychoneuroendocrinology.

[31] Hennebold JD, Daynes RA (1994). Regulation of macrophage dehydroepiandrosterone sulfate metabolism by inflammatory cytokines.. Endocrinology.

[32] Boulware DR, Hullsiek KH, Puronen CE, Rupert A, Baker JV (2011). Higher Levels of CRP, D-dimer, IL-6, and Hyaluronic Acid Before Initiation of Antiretroviral Therapy (ART) Are Associated With Increased Risk of AIDS or Death.. J Infect Dis.

[33] Van den Berghe G (2003). Endocrine evaluation of patients with critical illness.. Endocrinol Metab Clin North Am.

[34] Besedovsky HO, del Rey A (1996). Immune-neuro-endocrine interactions: facts and hypotheses.. Endocr Rev.

[35] Elenkov IJ, Chrousos GP (1999). Stress Hormones, Th1/Th2 patterns, Pro/Anti-inflammatory Cytokines and Susceptibility to Disease.. Trends Endocrinol Metab.

[36] Rook GA, Dheda K, Zumla A (2005). Immune responses to tuberculosis in developing countries: implications for new vaccines.. Nat Rev Immunol.

[37] Sakaguchi S, Miyara M, Costantino CM, Hafler DA (2010). FOXP3+ regulatory T cells in the human immune system.. Nat Rev Immunol.

[38] Rudensky AY (2011). Regulatory T cells and Foxp3.. Immunol Rev.

[39] Ribeiro-Rodrigues R, Resende Co T, Rojas R, Toossi Z, Dietze R (2006). A role for CD4+CD25+ T cells in regulation of the immune response during human tuberculosis.. Clin Exp Immunol.

[40] Toossi Z, Hirsch CS, Wu M, Mayanja-Kizza H, Baseke J (2011). Distinct cytokine and regulatory T cell profile at pleural sites of dual HIV/tuberculosis infection compared to that in the systemic circulation.. Clin Exp Immunol.

[41] Guyot-Revol V, Innes JA, Hackforth S, Hinks T, Lalvani A (2006). Regulatory T cells are expanded in blood and disease sites in patients with tuberculosis.. Am J Respir Crit Care Med.

[42] Horwitz DA (2010). Identity of mysterious CD4+CD25-Foxp3+ cells in SLE.. Arthritis Res Ther.

[43] Yang HX, Zhang W, Zhao LD, Li Y, Zhang FC (2009). Are CD4+CD25-Foxp3+ cells in untreated new-onset lupus patients regulatory T cells?. Arthritis Res Ther.

[44] Bonelli M, Savitskaya A, Steiner CW, Rath E, Smolen JS (2009). Phenotypic and functional analysis of CD4+ CD25- Foxp3+ T cells in patients with systemic lupus erythematosus.. J Immunol.

[45] Gavin MA, Torgerson TR, Houston E, DeRoos P, Ho WY (2006). Single-cell analysis of normal and FOXP3-mutant human T cells: FOXP3 expression without regulatory T cell development.. Proc Natl Acad Sci U S A.

[46] Bianchini R, Bistoni O, Alunno A, Petrillo MG, Ronchetti S (2011). CD4(+) CD25(low) GITR(+) cells: a novel human CD4(+) T-cell population with regulatory activity.. Eur J Immunol.

[47] Karagiannidis C, Akdis M, Holopainen P, Woolley NJ, Hense G (2004). Glucocorticoids upregulate FOXP3 expression and regulatory T cells in asthma.. J Allergy Clin Immunol.

[48] Coles AJ, Thompson S, Cox AL, Curran S, Gurnell EM (2005). Dehydroepiandrosterone replacement in patients with Addison’s disease has a bimodal effect on regulatory (CD4+CD25hi and CD4+FoxP3+) T cells.. Eur J Immunol.

[49] Perez AR, Bottasso O, Savino W (2009). The impact of infectious diseases upon neuroendocrine circuits.. Neuroimmunomodulation.

